# In Vivo Vascularization of Cell-Supplemented Spider Silk-Based Hydrogels in the Arteriovenous Loop Model

**DOI:** 10.3390/biomimetics10020117

**Published:** 2025-02-18

**Authors:** Justus Osterloh, Stefanie Heltmann-Meyer, Vanessa T. Trossmann, Aijia Cai, Yvonne Kulicke, Klara Terörde, Celena A. Sörgel, Isabell Lang, Harald Wajant, Thomas Scheibel, Tobias Fey, Dominik Steiner, Andreas Arkudas, Raymund E. Horch

**Affiliations:** 1 Department of Plastic and Hand Surgery, University Hospital of Erlangen, Friedrich-Alexander-University Erlangen-Nürnberg (FAU), 91054 Erlangen, Germany; 2 Department of Plastic and Hand Surgery, Medical Faculty of the University of Freiburg, University of Freiburg Medical Centre Freiburg, 79106 Freiburg, Germany; 3 Department of Biomaterials, University of Bayreuth, Prof.-Rüdiger-Bormann-Str. 1, 95447 Bayreuth, Germany; 4 Division of Molecular Internal Medicine, Department of Internal Medicine II, University Hospital Würzburg, 97070 Würzburg, Germany; 5 Bayreuth Center of Material Science and Engineering (BayMat), Universität Bayreuth, Universitätsstr. 30, 95440 Bayreuth, Germany; 6 Bavarian Polymer Institute (BPI), Universität Bayreuth, Universitätsstr. 30, 95440 Bayreuth, Germany; 7 Bayreuth Center of Colloids and Interfaces (BZKG), Universität Bayreuth, Universitätsstr. 30, 95440 Bayreuth, Germany; 8 Bayreuth Center of Molecular Bioscience (BZMB), Universität Bayreuth, Universitätsstr. 30, 95440 Bayreuth, Germany; 9 Faculty of Medicine, University of Würzburg, Pleicherwall 2, 97070 Würzburg, Germany; 10 Department of Materials Science and Engineering, Friedrich-Alexander-University Erlangen-Nürnberg (FAU), Martensstr. 5, 91058 Erlangen, Germany; 11 Department of Hand, Plastic, Reconstructive, and Burn Surgery, BG Trauma Clinic, University of Tübingen, 72076 Tübingen, Germany

**Keywords:** tissue engineering, vascularization, recombinant spider silk, reconstructive surgery

## Abstract

The goal of reconstructive surgery in treating tissue defects is to achieve a stable reconstructive outcome while minimizing donor site morbidity. As a result, tissue engineering has emerged as a key focus in the pursuit of this goal. One approach is to create a tissue container that can be preconditioned and later transplanted into the defect area. The characteristics of the matrices used in the tissue container are critical to this approach’s success. Matrices generated with recombinant, functionalized spider silk (eADF4(C16)-RGD) have been reported to be biocompatible and easy to vascularize. However, the effect of exogenously added proangiogenic cells, such as endothelial cells (T17b), on the vascularization process of matrices generated with this hydrogel in vivo has not been described yet. In this study, we implanted arteriovenous (AV) loop containers filled with a spider silk hydrogel consisting of an eADF4(C16)-RGD matrix and encapsulated, differentiated endothelial T17b cells producing the reporter protein TNFR2-Fc-Flag-GpL. The histological and µCT analyses revealed spontaneous angiogenesis and fibrovascular tissue formation in the container at 2 and 4 weeks post-implantation. The reporter protein was detected after 4 weeks. No severe immune response was observed. Altogether, this study demonstrates that cell-supplemented recombinant spider silk is a highly promising hydrogel to produce matrices for tissue engineering applications.

## 1. Introduction

Reconstructive surgery focuses on repairing the parts of the human body affected by congenital defects, trauma, or tumor resection [1]. Unfortunately, donor site morbidity at the location from which the tissues, grafts, or flaps are harvested is a major limitation [2,3,4]. Pain, discomfort, scarring, nerve or vessel damage, infection, delayed wound healing, aesthetic concerns, and functional limitations are just some of the potential risks that can occur at the donor site. Tissue engineering, as a field of regenerative medicine, is exploring the potential to minimize donor site morbidity in reconstructive surgery by providing alternative solutions to conventional tissue grafts or flaps [5]. The goal of tissue engineering is to create artificial constructs that mimic the structure and function of the tissue that needs to be replaced or reconstructed. Major challenges include low biocompatibility, poor biofunctionality, and a severe immune response. The fundamentals of tissue engineering involve the selection of a scaffold that provides optimal conditions for cell expansion and differentiation. More recently, with the advent of 3D bioprinting for the biofabrication of substitutes, tissue engineering has focused on hydrogels as ideal and printable matrices for tissue engineering purposes. Recombinant spider silk proteins, derived from the dragline silk of the European garden spider *Araneus diadematus*, are valid candidates to produce hydrogels for tissue engineering and biofabrication applications [6,7,8,9,10]. Recombinant spider silk materials exhibit low inflammatory responses and slow biodegradability [11,12]. The recombinant spider silk variant eADF4(C16) comprises 16 repeats of the C-module, mimicking the repetitive core domain of natural *Araneus diadematus* fibroin 4 [13]. Furthermore, eADF4(C16) has been genetically modified with the integrin-binding peptide RGD, which enhances its interaction with a multitude of cells [14,15,16]. Previously, it was shown that eADF4(C16)-based materials improve vascularization and de novo tissue formation in animal models [11,12,17]. However, the effect of proangiogenic cells on the behavior of recombinant spider silk hydrogels in terms of vascularization and biodegradation in vivo is unclear. T17b cells are known to promote angiogenesis and tissue formation [18,19,20]. Here, chemically transfected T17b cells expressing the reporter protein TNFR2-Fc-Flag-GpL were used as a model to analyze the spider silk hydrogels for their ability to act as scaffolds and to maintain cell activity. The TNFR2-Fc-Flag-GpL fusion protein itself consists of the extracellular domain of TNFR2, the Fc domain of human IgG1, and the luciferase of Gaussia princeps, serving as a reporter domain [21]. Previous studies have demonstrated the successful encapsulation and subsequent release of various proteins and drugs from recombinant spider silk hydrogels [21,22,23]. Furthermore, encapsulated cells were able to survive, grow, and maintain their functionality within spider silk hydrogels [9,21,24].

Another major challenge in tissue engineering is neovascularization, which is crucial for supplying oxygen and nutrients to the cells within constructs. The arteriovenous (AV) loop model of the rat is an excellent tool to study the vascularization of a scaffold in vivo [11,12,25,26,27]: By the interposition of a venous graft from the contralateral hind limb, an AV loop is formed by anastomosing the graft to the saphenous artery and vein. New vessels can sprout into the scaffolds from the loop (intrinsic vascularization), providing essential nutrients and ensuring the transplanted cells’ survival in the hydrogels. Another benefit is that the vascularized de novo tissue can be transplanted to a defect site by anastomosing the loop vessels to the recipient vessels. In a previous study, recombinant spider silk hydrogels were evaluated as a promising material for neovascularization in the AV loop without the addition of cells [12]. In this study, we implanted a polytetrafluorethylene (PTFE) chamber filled with a recombinant eADF4(C16)-RGD spider silk hydrogel containing reporter-protein-producing endothelial cells (T17b) into rats using the AV loop model. The purpose of this study was to analyze the effect of T17b cells on the neovascularization and matrix degradation of recombinant spider silk in the AV loop model of the rat to further elucidate the potential of recombinant eADF4(C16)-RGD spider silk hydrogels for tissue engineering.

## 2. Materials and Methods

### 2.1. T17b Cells with TNFR2-Fc-Flag-GpL

The endothelial progenitor cell (EPC) line T17b (murine) was cultured following established protocols [28]. The T17b cells were kindly gifted by Micheal Stürzl’s lab at the Division of Molecular and Experimental Surgery, Translational Research Center, Friedrich-Alexander-University Erlangen-Nürnberg (FAU). To generate stable T17b transfectants expressing TNFR2-Fc-Flag-GpL, the cells were transfected using polyethylenimin (PEI) [20]. In brief, for transfection in a 15 cm dish, 12 µg of a pCR3-based TNFR2-Fc-Flag-GpL-encoding expression plasmid was dissolved in 2 mL serum-free RPMI-1640 medium (Sigma-Aldrich, St. Louis, MO, USA) + 1% (*v*/*v*) Pen/Step, and then 36 µL of a 1 mg/mL PEI (Polyscience Inc., Warrington, PA, USA) solution was added dropwise under vortexing. The DNA/PEI mixture was incubated for 10 min at room temperature and was subsequently used together with 17 mL serum-free RPMI 1640 medium + 1% (*v*/*v*) Pen/Step to replace the medium in a 15 cm tissue culture petri dish with confluently grown T17b EPC. The following day, the serum-free medium was replaced by a new RPMI 1640 medium supplemented with 2% (*v*/*v*) fetal calf serum (FCS) + 1% (*v*/*v*) Pen/Step. After an additional day, selection with G418 (500 µg/mL) was started. After 2–3 weeks, when most of the cells were resistant, single clones were generated by limiting dilution and assayed to produce TNFR2-Fc-Flag-GpL.

### 2.2. T17b Cells Cultivation

The transfected, but undifferentiated, T17b endothelial progenitor cells were cultured in DMEM High Glucose, GlutaMAX (Gibco/Life Technologies, Waltham, MA, USA) supplemented with 10% (*v*/*v*) FCS (Merck, Burlington, MA, USA), 1% (*v*/*v*) Pen/Step (Gibco, USA), 1 mM Non-Essential Amino Acid (NEAA) (Gibco/Life Technologies), 2 mM HEPES buffer (pH 7.5, Gibco/Life Technologies), and 0.1 mM β-mercaptoethanol (Gibco/Life Technologies) at 37 °C under humidified conditions and 5% CO_2_. At least 72 h before use, the T17b cells were endothelially differentiated using DMEM supplemented with 1 μM all-trans-retinoic acid (ATRA; Sigma-Aldrich) and 0.5 mM adenosine 3′,5′-cyclic monophosphate (cAMP; Sigma-Aldrich).

### 2.3. Recombinant Spider Silk Hydrogel Generation

The recombinant spider silk protein eADF4(C16)-RGD consists of 16 repeats of a C module (sequence: GSSAAAAAAAASGPGGYGPENQGPSGPGGYGPGGP), which mimics the repetitive core sequence of the European garden spider’s (*Araneus diadematus*) dragline silk fibroin 4 (ADF4), and a C-terminal RGD integrin binding peptide was genetically fused to eADF4(C16) using an established cloning strategy [12,13]. The recombinant production in *Escherichia coli* BL21 gold (DE3) and the purification of eADF4(C16)-RGD (MW: 48.6 kDa) were performed as previously described [12,13]. The purity of the protein was ensured using Sodium Dodecyl Sulfate (SDS) polyacrylamide gel electrophoresis (SDS-PAGE) and a western blot of the *N*-terminally fused T7-tag as previously described [12,13]. The resulting protein was stored as lyophilized powder.

For the hydrogel preparation, eADF4(C16)-RGD powder was first dissolved in guanidinium thiocyanate (6 M; Carl Roth GmbH + Co. KG, Karlsruhe, Germany) at a concentration of 20 mg/mL for one hour at RT and sterile filtered (pore size 0.2 µm, Sartorius, Göttingen, Germany). A first dialysis using dialysis membranes with a MW-cutoff of 6–8 kDa (Thermo Scientific, USA) against 10 mM Tris/HCL (Carl Roth) was conducted to remove the denaturant (pH 7.5 overnight at room temperature) [11,20,23]. Afterwards, recombinant eADF4(C16)-RGD was concentrated to 35 mg/mL using polyethylene glycol dialysis (PEG, MW: 35,000 g/mol, Carl Roth) by the osmotic pressure-driven removal of water [11,20,23]. The concentration was monitored using a NanoDrop photo spectrometer (Thermo Fisher Scientific, Waltham, MA, USA). Due to the self-assembly of spider silk nanofibrils, a pre-gelled gel containing 3.5% (*w*/*v*) (35 mg/mL) spider silk was mixed with 15% (*v*/*v*) DMEM cell culture medium (see above) containing 1 × 10^7^ differentiated T17b cells to obtain a final gel concentration of 3% (*w*/*v*) in the hydrogel. For the in vitro experiments, 5 × 100 µL of the hydrogel were pipetted into a 96-well plate and covered with 100 µL DMEM medium after gelation at 37 °C (see below). For the in vivo experiments, 800 µL hydrogel was pipetted into a 1 mL syringe (B. Braun GmbH, Melsungen, Germany), gelled at 37 °C in a cell culture incubator overnight (HeraCell, Firma) and applied into the PTFE chamber during surgery.

### 2.4. Fluorescence and Confocal Laser Scanning Microscopy

A dead stain using ethidium homodimer-I (Eth-HD) (Invitrogen, Thermo Fisher Scientific, Erlangen, Germany) was conducted to assess cell viability and apoptosis of differentiated T17b cells inside the 3% (*w*/*v*) eADF4(C16)-RGD hydrogel after the overnight incubation in the syringe. Therefore, the gelled hydrogel was extruded from the syringe and stained using 4 μM Eth-HD in 1× PBS for 45–60 min in a humidified atmosphere (95% relative humidity, 5% CO_2_, 37 °C). Live staining using calcein acetoxymethyl ester was not possible due to the GFP fluorescence of the transfected T17b cells. Before imaging, the staining solution was exchanged using fresh 1× PBS. Fluorescence microscopy was conducted using a Leica DMI 3000B fluorescence microscope equipped with a 10× and 20× objective and the associated LAS X software version 3.7.2.22383 (Leica, Wetzlar, Germany). Z-stacks of hydrogels were recorded using a DMI 8 confocal laser scanning microscope (CLSM) equipped with a 10× objective and lasers using excitation wavelengths of 488 and 552 nm and the associated LAS X software version 3.5.7.23225 (Leica, Germany).

### 2.5. Cell Viability and Productivity Assays

The metabolic activity of the encapsulated T17b cells was assessed using the Colorimetric Cell Viability Kit I (WST-8, PromoCell, Heidelberg, Germany) following the manufacturer’s instructions. The viability assay was performed on days 1, 3, 7, 10, and 14 to evaluate the viability and proliferation of T17b cells. Next, a 10% (*v*/*v*) WST-8 solution was added to the cell culture medium and incubated for two hours at 37 °C. Subsequently, 100 µL of the supernatant was transferred into a black 96-well plate (Greiner Bio-One, Kremsmünster, Austria), and the absorbance was measured at 450 nm using a microplate reader (Thermo Fisher Scientific). The release of TNFR2-Fc-GpL from the cells was determined by measuring the GpL luciferase activity of TNFR2-Fc-GpL in the supernatant after 1, 3, 7, 10, and 14 days. For this purpose, 10 µL of the supernatant was taken and mixed with 90 µL of a DMEM medium containing 0.5% (*v*/*v*) FCS and 1% (*v*/*v*) Pen/Strep, and the relative light units (RLUs) were determined using a luminometer (Berthold Technologies, Bad Wildbad, Germany), as described by Trossmann et al. [20].

### 2.6. Arteriovenous Loop Surgery

For each group (eADF4(C16)-RGD 2-week explantation group; eADF4(C16)-RGD 4-week explantation group), 6 male Lewis rats underwent microscopy-based arteriovenous loop surgery, as described previously [10,11,25,26]. During the surgical interventions, anesthesia was applied using isoflurane under spontaneous breathing. After an incision on the left inner thigh, the saphenous artery and vein were dissected. The loop was created by taking a vein graft from the contralateral thigh (saphenous vein). Venous–venous and arteriovenous anastomoses were performed with 11-0 Ethilon (Ethicon, Raritan, NJ, USA). A (PTFE) chamber (10 × 5 mm) was half filled with the spider silk hydrogel, including 1 × 10^7^ T17b cells per 1 mL of eADF4(C16)-RGD, and the chamber was placed into the left thigh of the rat. The AV loop was then placed in the chamber. Following, the chamber was filled with the remaining eADF4(C16)-RGD spider silk hydrogel. Afterwards, the chamber was closed with a lid and sutured to the underlying muscle, and the skin was closed and sutured. The animals received pain medication and antibiotics for one week postoperatively. Approval for the animal experiments (Reference Number: AZ 55.2-2532-2-763) was obtained from both the Animal Care Committee of the University of Erlangen and the Mittelfranken Government. The experiments were conducted in compliance with the EU Directive 2010/63/EU and are reported in accordance with ARRIVE guidelines.

### 2.7. Explantation Procedure

Two and four weeks after implantation, the constructs were explanted as previously described [10,11]. Before explantation, the animals were perfused with contrast agent Microfil^®^ (Flow Tech, Inc., South Windsor, CT, USA) for the further analysis of blood vessel formation with a µCT analysis. The animals were first systemically perfused with a warm heparin solution via the aorta. Thereafter, the contrast agent Microfil^®^ was applied. The contrast agent was prepared according to the manufacturer’s instructions. The explanted construct was fixed in formalin (Histofix 4%, Carl Roth) for 24 h and then stored in Dulbecco’s Phosphate Buffered Saline (DPBS, Sigma-Aldrich) until further use.

### 2.8. Micro Computed Tomography (µCT)

The µCT scans were performed using Skyscan 1172 with an 11 MP detector and a tungsten tube at a voltage of 80 kV and a current of 100 mA (Skyscan B.V., Leuven, Belgium), as described previously [11]. After scanning at 180° with a rotation step of 0.25° and a resolution of 4.47 μm/voxel, the data were reconstructed with Radeon back transformation using the tomographic reconstruction software (NRecon Client and Server 1.7.42 with GPU support; Skyscan) while adjusting the X/Y shift and alignment during measurement. The visualization and vessel radius evaluation were carried out by imaging software (Amira 2021.1; Thermo Fisher Scientific).

### 2.9. (Immuno-)Histological Staining

After completing the µCT scans, the constructs were embedded in paraffin and cut into 3 µm slices. Hematoxylin and eosin (H&E), as well as α-smooth muscle actin (α-SMA, Zytomed Systems GmbH, Berlin, Germany) staining, were prepared according to the standard protocols [10,11]. Biocompatibility was assessed with CD68 staining to detect macrophages. After deparaffinization and rehydration, the samples were incubated with pronase (AppliChem GmbH, Darmstadt, Germany) at room temperature for 20 min. Then, the samples were blocked with blocking solution (Zytomed Systems GmbH) and incubated with the primary antibody (anti-CD68, BIO-RAD, Hercules, CA, USA; dilution 1:300) overnight at 4 °C. After washing, the slices were incubated with the secondary antibody (alkaline phosphatase-labeled anti-mouse antibody, for 30 min) and stained with Fast Red TR/Naphthol AS (both Sigma-Aldrich). Counterstaining was performed using hemalum (Merck). The Flag-tag staining was performed using the ZytoChem Plus HRP Polymer system with the recombinant Anti-DDDDK-tag (Abcam, Cambridge, UK, dilution 1:750) as the primary antibody. After washing and peroxide blocking, the secondary antibody Dako REAL EnVision HRP Rabbit/Mouse (Agilent Technologies, Santa Clara, CA, USA) was applied. Counterstaining was also performed with hemalum. For the further analysis, images of the sections were either taken with the Olympus IX81 microscope (Olympus, Hamburg, Germany) using the cellSens Dimension V1.5 software of Olympus or scanned with the PANNORAMIC 250 Flash scanner (3DHISTECH, Budapest, Hungary), and the images were prepared using CaseViewer 2.4 (3DHISTECH).

### 2.10. Statistical Analysis

The results are graphically presented as mean ± standard deviation. Cell viability rates were determined by quantifying live and dead cells from confocal images using imageJ version 2. The statistical analysis was performed using GraphPad Prism 10.00 (GraphPad Software, San Diego, CA, USA). The data were first tested for normal distribution and then analyzed for statistical significance using a *t*-test after the verification showed that all the data were normally distributed. A critical level of statistical significance was set at *p* ≤ 0.05.

## 3. Results

### 3.1. In Vitro Evaluation

**Figure 1:**
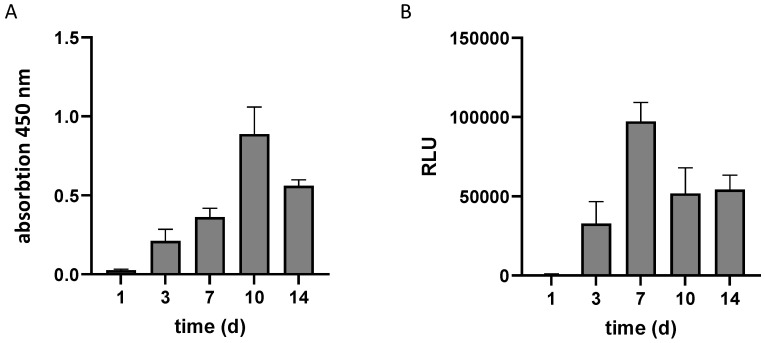
The in vitro analysis of (**A**) cell proliferation (WST-8-assay) and (**B**) productivity (luciferase assay) of transfected T17b cells in an eADF4(C16)-RGD spider silk scaffold (*n* = 4).

**Figure 2:**
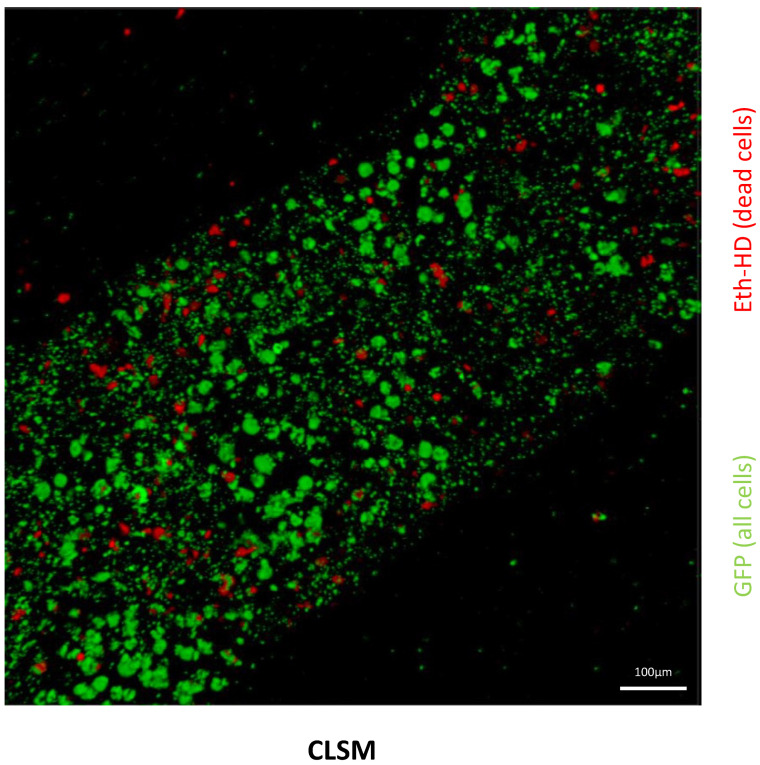
The confocal laser scanning microscopy (CLSM) of transfected T17b cells within an eADF4(C16)-RGD spider silk scaffold also indicates that the majority of cells remain viable following overnight incubation. All cells display GFP fluorescence, while dead cells are red, based on ethidium homodimer-I staining (Eth-HD) (scale bar: 100 µm).

To evaluate the ability of T17b cells to proliferate and to produce the reporter protein TNFR2-Fc-Flag-GpL fusion protein in an eADF4(C16)-RGD spider silk scaffold in vitro, a WST-8-assay and a luciferase assay were performed. The WST-8-assay of T17b cells in a spider silk scaffold, as an indicator of cell proliferation, showed an increase in absorption on days 1, 3, and 7, reaching a maximum on day 10 (Figure 1). The cells in the matrix furthermore stably produced the luciferase reporter, which was detectable in the supernatant via a luciferase assay over the entire time, with a maximum on day 7. Furthermore, cell viability was evaluated using confocal laser scanning microscopy. Following the overnight incubation of transfected T17b cells in the eADF4(C16)-RGD spider silk scaffold, only a small number of cells tested positive for the staining of dead cells (Eth-HD) (Figure 2). The quantitative analysis of cell viability in the CLSM images showed that the average survival rate was 94.5 ± 0.7%.

### 3.2. AV Loop Surgery and Postoperative Outcome

**Figure 3:**
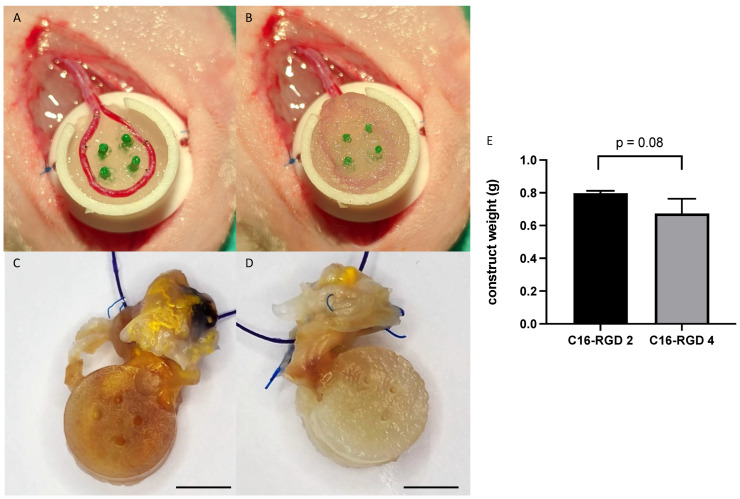
Images during the AV loop surgery: A 1 cm diameter PTFE chamber is half filled with the hydrogel, and the AV loop is placed into the chamber on the hydrogel surface. The green columns provide support to stabilize the AV loop and maintain its position (**A**). Afterwards, the AV loop is covered with hydrogel until the chamber is filled completely with hydrogel (**B**). Images of the explants 2 weeks (**C**) and 4 weeks (**D**) after implantation show a stable construct and yellow color around the entrance of the chamber due to the perfusion with Microfil^®^. Scale bar: 0.5 cm. No significant differences were found between construct weights after 2 (C16-RGD 2) and 4 (C16-RGD 4) weeks of implantation (**E**).

The AV loop model of the rat was used to assess the vascularization potential of the eADF4(C16)-RGD spider silk scaffold in the presence of proangiogenic T17b endothelial cells in vivo. All the animals tolerated the surgery well and showed no signs of hematoma, seroma, or infection. For each group, three specimens (2-week explantation group 3/6 = 50%; 4-week explantation group 3/6 = 50%) showed patent AV loops after explantation (Figure 3A–D). For further analysis, the samples from those animals were used, while all other samples were excluded. Macroscopically, there were no indications of a foreign-body reaction due to the implanted PTFE chamber. After explantation, there was no significant difference in the construct weight between the 2-week explantation group and the 4-week explantation group (2 weeks 0.797 ± 0.015 g; 4 weeks 0.673 ± 0.091 g; *p* = 0.08) (Figure 3E).

### 3.3. Histology and Quantification

**Figure 4:**
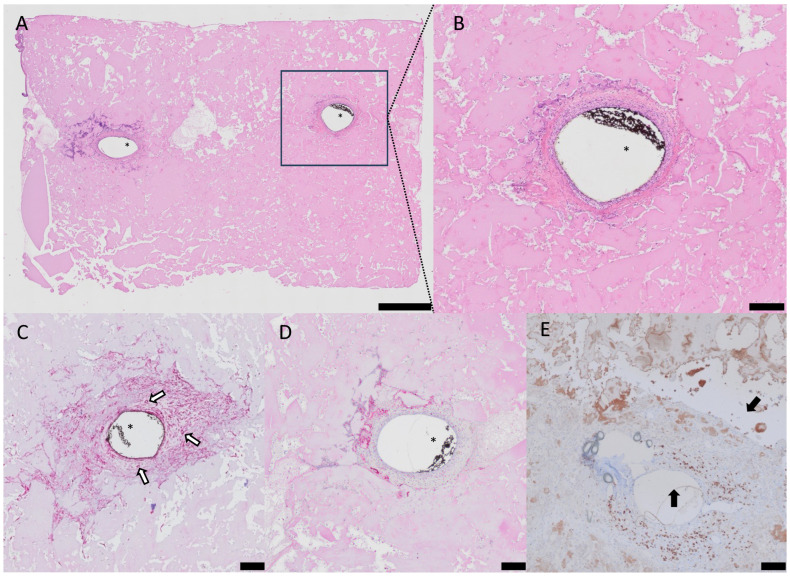
H&E staining was performed to provide a histological overview, with (**A**) displaying an overview image with the AV Loop on the left and right sides, surrounded by newly formed tissue, while (**B**) offers more detailed views of the eADF4(C16)-RGD hydrogel after a 2-week period. α-SMA staining (**C**) (white arrows point at small vessels) showed the neovascularization of small vessels surrounding the AV loop, while staining with an anti-CD68 antibody (**D**) revealed the presence of only a few lymphocytes after a 4-week duration. Flag-tag staining (brown) showed the presence of the reporter protein around the AV loop 4 weeks after implantation ((**E**), black arrows point at reporter protein expression). Note the presence of black Microfil^®^ inside the vessel lumen (*). Scale bars are indicated as 500 µm for (**A**) and 200 µm for (**B**–**E**).

The patent AV loop samples were further histologically analyzed to assess neovascularization, biocompatibility, and localization and the distribution of the reporter protein TNFR2-Fc-Flag-GpL. In the patent AV loop samples, the vessels were filled with Microfil^®^, while no perfusion medium was found in the extravascular compartments. Newly built fibrovascular tissue and infiltrating cells were detected around the loop vessels and not in the periphery of the constructs (Figure 4A–D). To evaluate the biocompatibility of the eADF4(C16)-RGD hydrogels with encapsulated TNFR2-Fc-Flag-GpL-producing T17b cells, CD68 staining was conducted (Figure 4D). In all the samples, migrating macrophages around the AV loops could be detected, but no multinuclear giant cells or extensive lymphatic invasion of cells were found. Furthermore, there was no difference in the distribution or count of immune cells between the groups. The reporter protein TNFR2-Fc-Flag-GpL, which is produced and secreted from T17b cells, could be stained with anti-Flag antibody staining. Anti-Flag signals were found predominantly near the AV loop vessels, but not in the periphery (Figure 4E).

**Figure 5:**
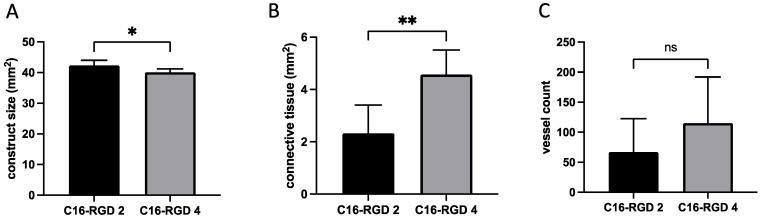
Quantification of histological findings: Construct size decreased significantly (**A**), while newly formed connective tissue increased significantly over time (**B**). Vessel count did not increase significantly when comparing 4 weeks and 2 weeks after implantation (**C**). (ns = not significant; * *p* ≤ 0.05; ** *p* ≤ 0.01).

The H&E staining suggests a modest degradation of the spider silk matrix at the time of explantation. The construct size decreased significantly after 4 weeks compared to 2 weeks after implantation (2 weeks 42.32 ± 1.570 mm^2^; 4 weeks 40.06 ± 1.114 mm^2^; *p* = 0.02; *) but, in total, only to a moderate degree of 5.34% (Figure 5A). More importantly, the eADF4(C16)-RGD spider silk constructs exhibited the progressive de novo generation of newly formed tissue over time (2 weeks 2.33 ± 1.08 mm^2^; 4 weeks 4.57 ± 0.94 mm^2^; *p* = 0.004; **) (Figure 5B). The α-SMA staining showed substantial neovascularization around the AV loop in all the samples, but there was no significant additional increase in the vessel count during the 2- to 4-week period (2 weeks 67 ± 55; 4 weeks 115 ± 77; *p* = 0.43) (Figure 5C).

### 3.4. µCT Analysis

**Figure 6:**
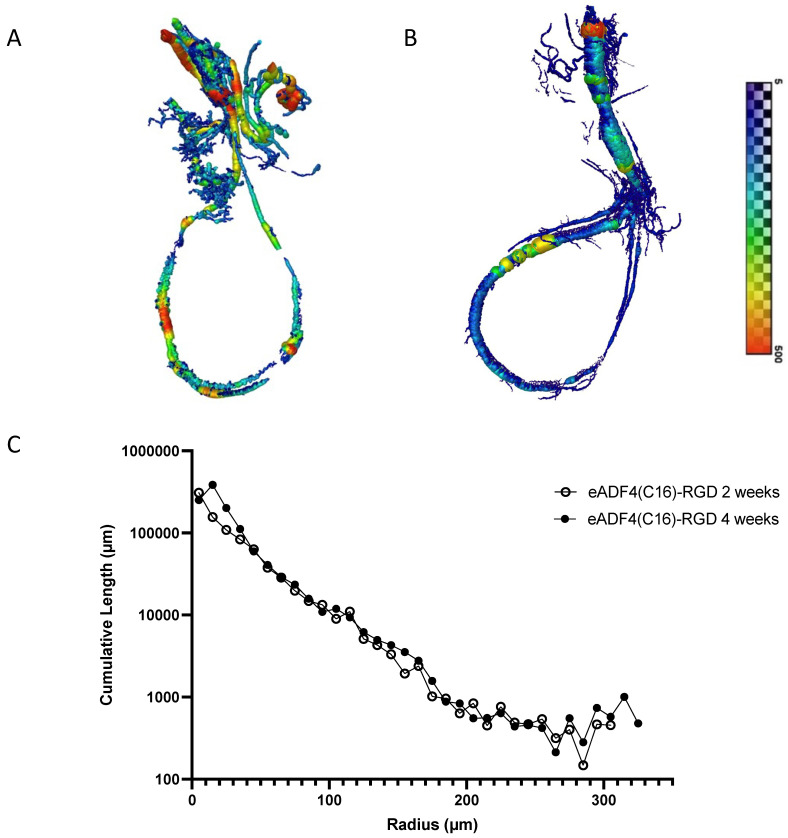
The AV loops were perfused with Microfil^®^ and analyzed using µCT. Image reconstruction of µCT scans 2 (**A**) and 4 (**B**) weeks after the implantation of eADF4(C16)-RGD hydrogels in the presence of T17b cells. The scale bar depicts the mean vessel radius in micrometers. The numeric colors represent the vessel radius from small (blue, 5 µm) to large (red, 500 µm). The quantitative analysis of the cumulative length relative to the vessel radius (µm) (**C**).

To further elucidate the quantification and distribution of newly formed vessels in the eADF4(C16)-RGD spider silk scaffolds of the specimens with patent AV loops, µCT scans were performed. The analysis of three-dimensional µCT scans confirmed the patency of the AV loops, as seen in the histological staining (2-week explantation group 3/6 = 50%; 4-week explantation group 3/6 = 50%). Sprouting vessels from the AV loop could be detected after 2 weeks of implantation. After 4 weeks, the vessel network showed a trend to increase (Figure 6A,B). There was no significant difference in the cumulative length of the vessels or in the distribution of the vessel radius relative to the cumulative length when comparing the 2- and 4-week explantation groups (Figure 6C).

## 4. Discussion

Due to their unique mechanical and biological properties, eADF4(C16)-RGD hydrogels represent a promising biomaterial for tissue engineering applications [12,21]. The capacity of biomaterials to promote vascularization for adequate oxygen and nutrient delivery is critical for potential clinical applications. The AV loop model of the rat is an ideal approach to studying the intrinsic vascularization of scaffolds in vivo. Previous studies have shown that eADF4(C16)-based matrices have good biocompatibility and are easy to vascularize [11,12], but less is known about the influence of proangiogenic cells on recombinant spider silk (eADF4(C16)-RGD) based-hydrogels and the ability of these hydrogels to support the cell survival of encapsulated cells in vivo. In this study, endothelial cells (T17b) were embedded into the spider silk hydrogels and transplanted into the groin of Lewis rats using the AV loop model. After explantation, we found a patency rate of 50%, which did not differ between the 2- and 4-week explantation groups. In addition to neovascularization, the remodeling of bioengineered scaffolds from artificial hydrogel tissue to host tissue is a critical feature of biomaterials. Ideally, the scaffold should degrade while simultaneously promoting the formation of newly formed tissue. In the recombinant eADF4(C16)-RGD spider silk scaffolds, biodegradation occurred slowly, and newly formed tissue was built over time. While the weights of the constructs did not differ significantly, the histological analysis revealed that the size of the eADF4(C16)-RGD scaffolds decreased significantly. In contrast to the study by Steiner et al. [12], in which recombinant spider silk hydrogels without proangiogenic cells were implanted using the AV loop model and a significant enlargement of the vessel network was reported 4 weeks after implantation, we could not show a significant growth of the vessels at 4 weeks compared to 2 weeks after implantation (Figure 5 and Figure 6). This may be due to the proangiogenic effect of the T17b cells, resulting in accelerated initial growth and an earlier plateau of vascular network development. It would be interesting to determine if extending the implantation period to 8 or even 12 weeks would reveal significant differences in the vascularization and immune response in the recombinant spider silk (eADF4(C16)-RGD) scaffolds. In tissue engineering, the role of growth factors is another critical aspect of enhancing angiogenesis. It has been proposed to use growth factors like basic fibroblast growth factor (bFGF) or vascular endothelial growth factor (VEGF) in the AV loop model [29]. Although these growth factors promote angiogenesis from existing blood vessels, they can only slightly enhance the natural growth rate of blood vessels [30]. Moreover, in this study, only one cell line (T17b cells) was investigated. It should be noted that the source and characteristics of the cells used in tissue engineering approaches also have a tremendous impact on the outcome. In addition, parameters such as cell density and the inclusion of multiple cell types could further influence tissue vascularization and scaffold remodeling. Exploring these factors in future studies would provide a more comprehensive understanding of the interplay between the eADF4(C16)-RGD scaffold and the cellular environment, potentially enhancing the applicability of this hydrogel for tissue engineering purposes. In the present study, biocompatibility testing showed no evidence of massive macrophage invasion or an increased presence of multinuclear giant cells, two indicators of severe immunoreactivity, suggesting the good biocompatibility of cell-supplemented recombinant eADF4(C16)-RGD spider silk matrices. These results are consistent with previous in vivo studies of implanted cell-free spider silk matrices [31]. However, a limitation of this study is that the data presented provide only a broad overview of matrix biocompatibility, focusing on specific subpopulations of lymphocytes. A more detailed characterization and quantification of lymphocytes would be required for a deeper understanding of the immune response. In a study conducted by Trossmann et al. [21], eADF4(C16)-RGD hydrogels were loaded with HEK293 producer cells that were stably secreting the traceable reporter TNFR2-Fc-Flag-GpL. It was demonstrated that eADF4(C16)-RGD hydrogels are a suitable carrier matrix for genetically modified cells producing biologicals, and that the latter are rapidly released from scaffolds printed with this type of hydrogel in a functional form [21]. Hence, our initial goal was to transplant protein-secreting cells into the AV loop model of the rat to demonstrate that the reporter protein could be released and function in vivo. Our in vitro experiments demonstrated the viability of the T17b cells in the gel, showing that they survive the process of encapsulation in the spider silk hydrogel. The cells also produced the reporter molecule, which was detectable in the supernatant throughout the experiment (Figure 1 and Figure 2). While the viability of various encapsulated cell types (HEK293 cell line, BxPC-3 epithelial pancreas cell line (ATCC CRL-1687), mouse fibroblast cell line BALB/3T3) in spider silk-based hydrogels were shown in vitro in previous studies [9,21,32], this is the first to demonstrate their viability and functionality in vivo using the arteriovenous loop model. Unfortunately, despite our efforts, we could not detect the reporter protein through a luciferase assay in any of the animals by analyzing blood and urine samples at any point in time. A possible explanation for this observation is that the productivity of the T17b producer cells resident in the matrix was not sufficiently high enough to allow for the detection of the biological reporter protein in the circulation or urine after release. In vitro data show that HEK293 cells proliferate significantly faster and produce significantly more fusion protein than T17b cells [21]. However, it should be noted that immunological effects must be expected when implanting the human HEK293 cell line into immunocompetent rats. Another plausible reason for our inability to detect the reporter protein in the circulation could be due to the limited number of viable cells present in the steady state reached in the matrix after 2–4 weeks. Indeed, we were able to detect the reporter protein TNFR2-Fc-GpL by IHC preferentially in the cells around the AV loop, where nutrient availability is high, but not at the periphery of the matrix. In vitro data indicate that nutrient transport through eADF4(C16)-RGD hydrogels is possible, even for high molecular weight proteins [32]. However, in the in vivo scenario, the nutrient supply appears insufficient for encapsulated cells at the periphery of the construct. Therefore, an alternative approach could involve positioning the cells within the gel exclusively near the loops. Overall, further adjustments to the experimental setup are required to demonstrate the achievement of steady-state numbers of drug-producing cells in the AV loop chamber and ensure the presence of the reporter protein in the blood at therapeutically relevant concentrations. In addition to spider silk-based scaffolds, several other matrices have been extensively studied for tissue engineering purposes, as well as for their potential for neovascularization using the AV loop model. Similar to spider silk-based hydrogels, hydrogels made of fibrin [20,25,33] or alginate [34] have been evaluated in the rat AV loop model and have also demonstrated scaffold vascularization. Fibrin-based hydrogels with low concentrations of fibrin have been shown to be unstable constructs as they are rapidly degraded by fibrinolytic enzymes. While increasing the concentration of fibrin or incorporating antifibrinolytics can slow this degradation, these approaches significantly inhibit neovascularization and tissue growth [25,33,35]. Pure alginate-based hydrogels display dense structures with low biodegradability and high stiffness. However, modified alginate-based hydrogels, such as ADA-GEL, have demonstrated promising outcomes in terms of cytocompatibility, vascularization, and biodegradability [34]. As a result, the choice of hydrogel composition for studying neovascularization in the AV loop model depends heavily on the specific scientific objectives. This experimental setup’s clinical application could be significant, as a transplantable tissue container with effector protein-producing cells in a hydrogel could also be transplanted into human patients using the AV loop model. For instance, cells that produce insulin in people with diabetes, or cells that produce recombinant proteins and antibodies that act as immune system inhibitors, could be used to treat patients with autoimmune diseases or cancer. In clinical scenarios, a unique potential advantage of this very specific model is the easy removal of the therapeutic container in case of overproduction. The clinical potential of the AV loop model in a different context was demonstrated by Horch et al. [36], who successfully utilized the AV loop model to create axially vascularized large bone grafts for treating bone defects following debridement and osteomyelitis in two patients. However, there are still limitations regarding the applicability of the AV loop model to humans, as large-scale clinical trials are currently lacking. Admittedly, standardized clinical application is still a long way off, and further research is needed to make progress toward the implementation of tissue engineering using the AV loop model in clinical practice.

## 5. Conclusions

In the present study, we demonstrated the intrinsic vascularization of eADF4(C16)-RGD spider silk-based matrices supplemented with proangiogenic endothelial cells (T17b) in the arteriovenous loop model of the rat without detecting strong immunoreactivity or the release of a functional reporter protein. Since high biocompatibility, the ability to encapsulate viable and functional cells, and the formation of new blood vessels are key features for artificial tissue culture matrices, eADF4(C16)-RGD spider silk-based hydrogels are excellent candidates for such tissue engineering purposes. The combination of recombinant spider silk matrices and producer cells is a promising approach to generating bioartificial tissues in the future.

## Data Availability

Data supporting the findings of this study are available within the paper.
